# Supplementary material for: Genomic retargeting of p53 and CTCF is associated with transcriptional changes during oncogenic HRas-induced transformation
Source: Commun Biol. 2020 Nov 25;3:696. doi: 10.1038/s42003-020-01398-y (PMC7809021; doi:10.1038/s42003-020-01398-y)
Supplement: Supplementary file 1 — Supplementary Information [file 42003_2020_1398_MOESM1_ESM.pdf]

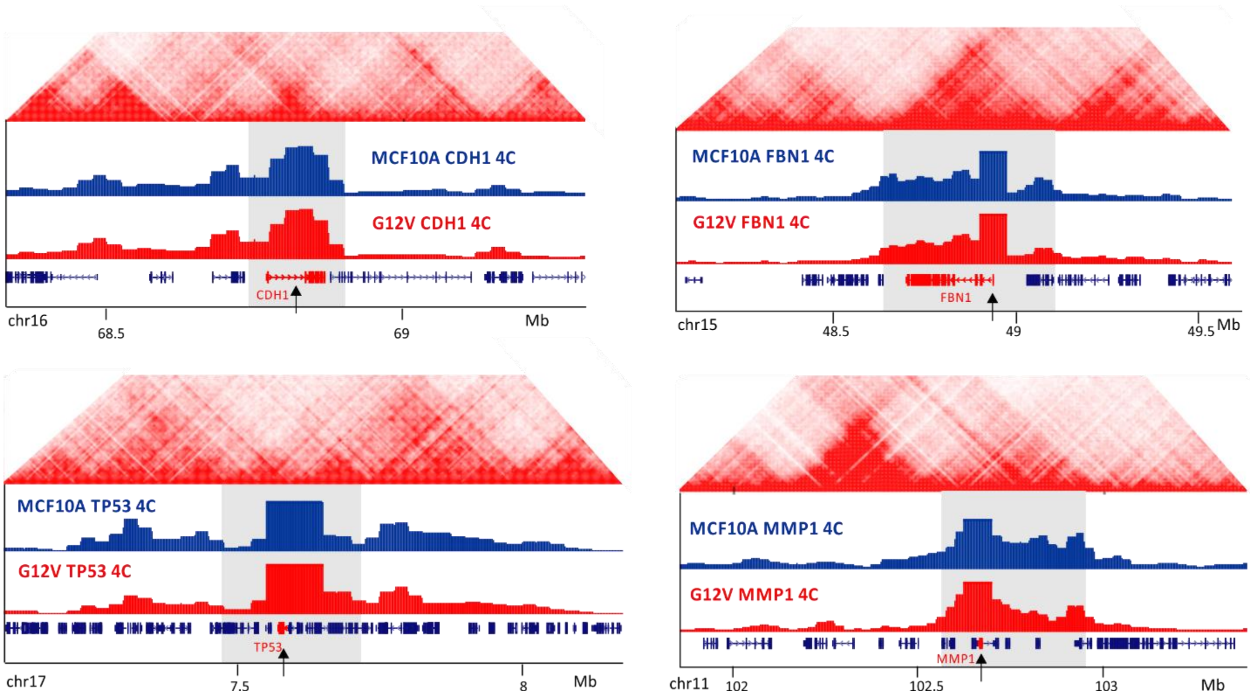

Supplementary Figure S1. 4C profiles. Four examples of 4C-seq profiles: *CDH1* (down-regulated gene), *FBN1* (up-regulated gene), *TP53* (unchanged gene) and *MMP1* (low expressing gene), in MCF10A and G12V MCF10A cells. Domains are marked with gray box, genes marked with black arrow. Hi-C data from GM12878 is shown on the top [23]. Chromosomal coordinates in Mb of human hg19 genome build are indicated in the bottom.

A

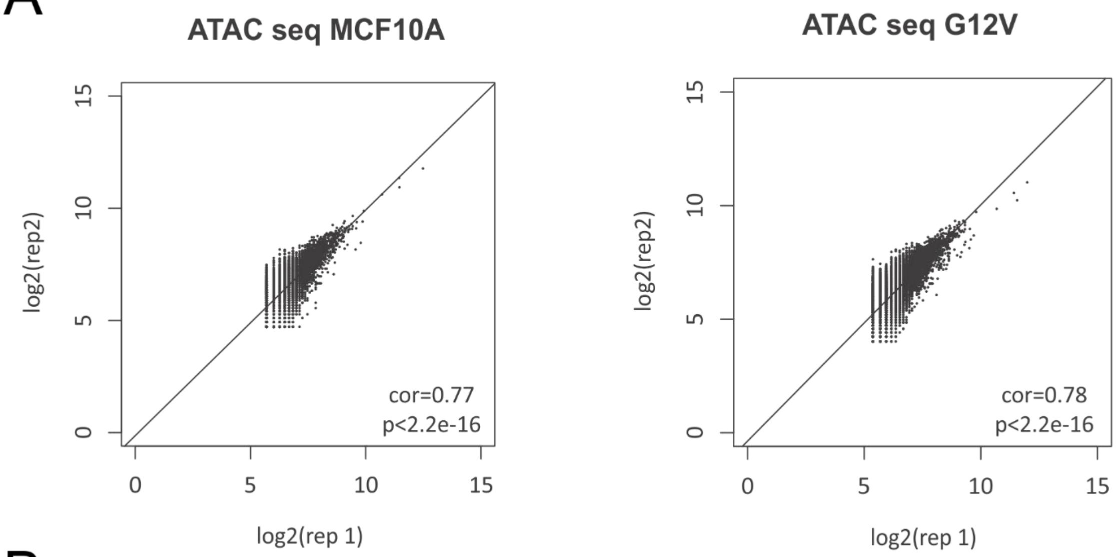

B

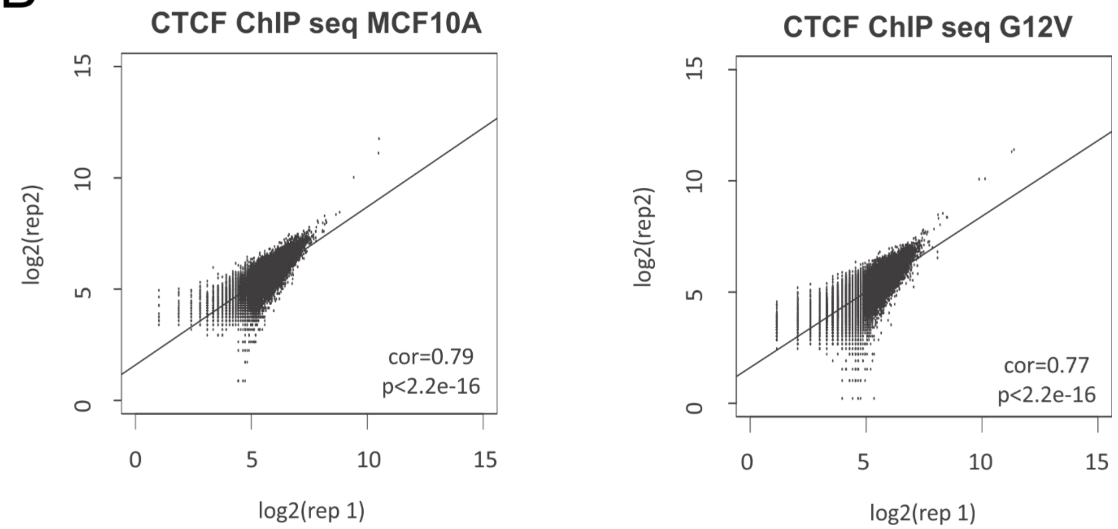

C

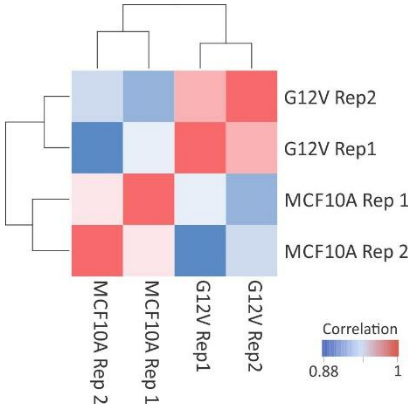

Supplementary Figure S2. RNA-seq, **ATAC-seq and ChIP-seq reproducibility**. Correlation plots between ATAC-seq replicas (A) and ChIP-seq replicas (B). log2CPM of replica 1 within peaks was plotted against the log2CPM of replica 2 within peaks. Values for Pearson correlation and test are shown. (C) hierarchical clustering of the pearson correlation between the RNA-seq replicas.

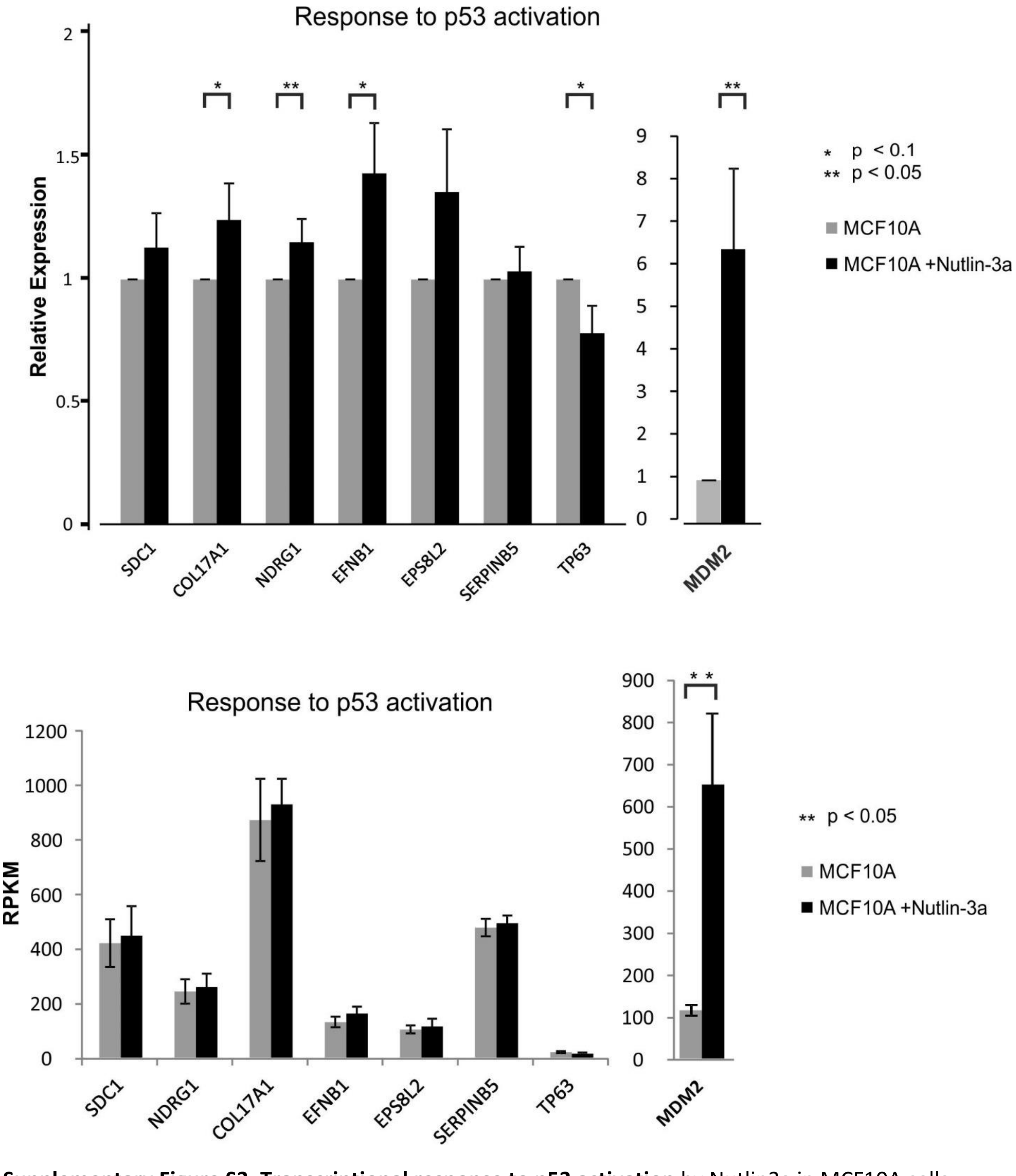

Supplementary Figure S3. Transcriptional response to p53 activation by Nutlin3a in MCF10A cells measured by real time PCR (top) and RNA-seq (bottom). genes in this figure were down regulated after HRas-induced transformation and contain p53 motif in their lost DARs. p-students t test and SD of three replicas are presented.

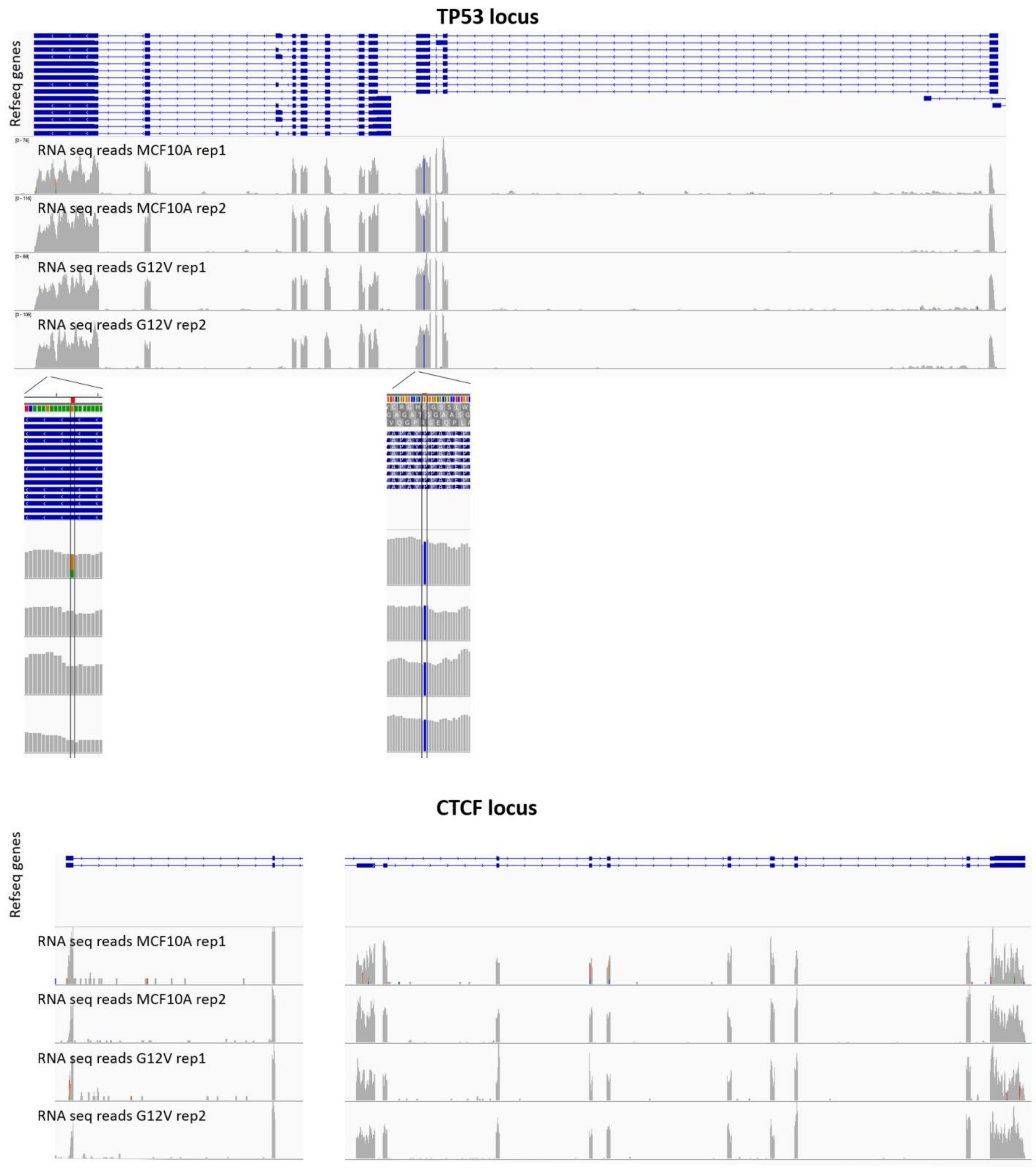

Supplementary Figure S4. Sequence integrity of *TP53* and *CTCF*. IGV browser shots of RNA-seq reads from MCF10A and G12V cells mapped to *TP53* and *CTCF* loci. Two replicas are shown. Gray color indicates agreement with the human reference genome (hg19) sequence. Other colors are present in low proportion in individual replicas indicate sequencing errors. The deviation from hg19 sequence in *TP53* from CCC to CGC in exon 4 exchange proline to arginine at codon 72 of p53. This is a known variation that occurs at a high frequency (>50%) in some populations (Pietsch et al., PMID: 16550160).

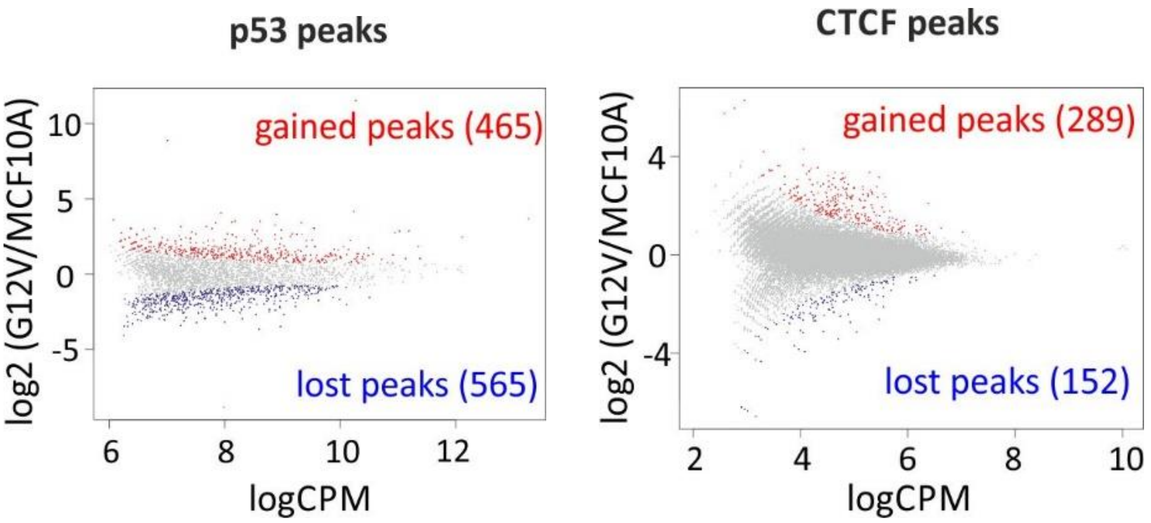

Supplementary Figure S5. Differential p53 and CTCF binding. MA plot displaying the mean normalized counts (Counts Per Million (CPM), x-axis) versus the log 2 fold change between MCF10A and G12V cells (y-axis), of the p53 ChIP-seq (left) and CTCF (right) peaks. Red and blue points represent unique G12V and unique MCF10A peaks, respectively, as declared by EdgeR analysis.

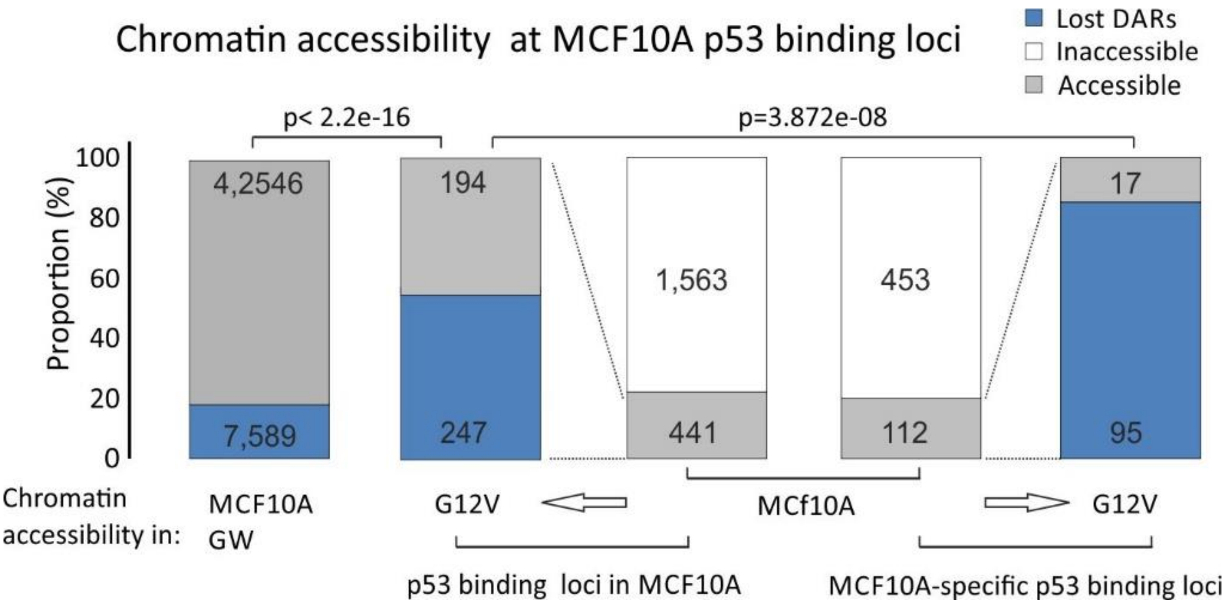

Supplementary Figure S6. Chromatin accessibility at MCF10A p53 binding loci. The number of p53 ChIP peaks in MCF10A is indicated. The chromatin accessibility state of these loci in MCF10A and G12V is color coded. p-proportional test.

Motif enrichment in G12V-specific p53 ChIP peaks

Total Target Sequences = 465, Total Background Sequences = 48020

Motif	Name	P-value	log P-pvalue	q-value (Benjamini)	# Target Sequences with Motif	% of Targets Sequences with Motif	# Background Sequences with Motif	% of Background Sequences with Motif
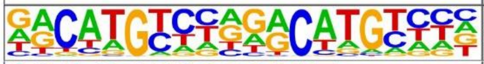	p53(p53)/Saos-p53-ChIP-Seq(GSE15780)/Homer	1e-478	-1.102e+03	0.0000	320.0	68.82%	485.0	1.01%
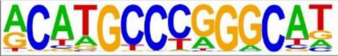	p53(p53)/mES-cMyc-ChIP-Seq(GSE11431)/Homer	1e-106	-2.456e+02	0.0000	81.0	17.42%	134.2	0.28%
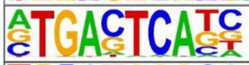	AP-1(bZIP)/ThioMac-PU.1-ChIP-Seq(GSE21512)/Homer	1e-38	-8.939e+01	0.0000	111.0	23.87%	2609.3	5.41%
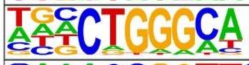	ZNF416(Zf)/HEK293-ZNF416.GFP-ChIP-Seq(GSE58341)/Homer	1e-23	-5.492e+01	0.0000	196.0	42.15%	10134.5	21.01%
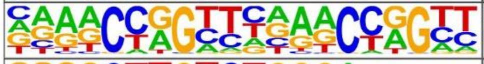	Tcfcp2l1(CP2)/mES-Tcfcp2l1-ChIP-Seq(GSE11431)/Homer	1e-17	-4.133e+01	0.0000	37.0	7.96%	579.2	1.20%
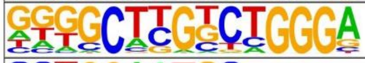	Zfp809(Zf)/ES-Zfp809-ChIP-Seq(GSE70799)/Homer	1e-15	-3.632e+01	0.0000	57.0	12.26%	1624.9	3.37%
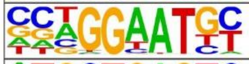	TEAD4(TEA)/Tropoblast-Tead4-ChIP-Seq(GSE37350)/Homer	1e-11	-2.723e+01	0.0000	83.0	17.85%	3735.6	7.74%
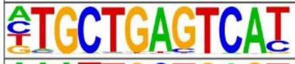	Nrf2(bZIP)/Lymphoblast-Nrf2-ChIP-Seq(GSE37589)/Homer	1e-10	-2.365e+01	0.0000	16.0	3.44%	173.2	0.36%
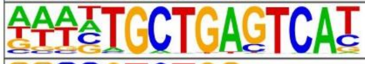	Bach1(bZIP)/K562-Bach1-ChIP-Seq(GSE31477)/Homer	1e-7	-1.839e+01	0.0000	13.0	2.80%	155.6	0.32%
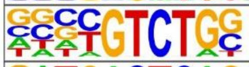	Smad4(MAD)/ESC-SMAD4-ChIP-Seq(GSE29422)/Homer	1e-6	-1.578e+01	0.0000	124.0	26.67%	8206.9	17.01%
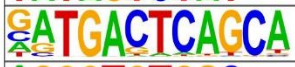	NF-E2(bZIP)/K562-NFE2-ChIP-Seq(GSE31477)/Homer	1e-5	-1.365e+01	0.0000	12.0	2.58%	201.7	0.42%
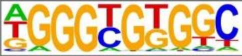	KLF5(Zf)/LoVo-KLF5-ChIP-Seq(GSE49402)/Homer	1e-5	-1.333e+01	0.0000	128.0	27.53%	8951.5	18.56%
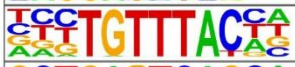	FOXP1(Forkhead)/H9-FOXP1-ChIP-Seq(GSE31006)/Homer	1e-5	-1.234e+01	0.0001	32.0	6.88%	1342.4	2.78%
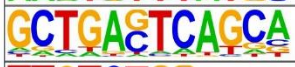	MafK(bZIP)/C2C12-MafK-ChIP-Seq(GSE36030)/Homer	1e-5	-1.167e+01	0.0001	25.0	5.38%	940.2	1.95%
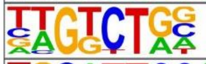	Smad3(MAD)/NPC-Smad3-ChIP-Seq(GSE36673)/Homer	1e-4	-1.096e+01	0.0003	176.0	37.85%	13886.7	28.79%
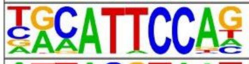	TEAD3(TEA)/HepG2-TEAD3-ChIP-Seq(Encode)/Homer	1e-4	-1.067e+01	0.0003	72.0	15.48%	4538.9	9.41%
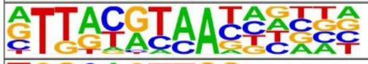	NFIL3(bZIP)/HepG2-NFIL3-ChIP-Seq(Encode)/Homer	1e-4	-1.010e+01	0.0006	39.0	8.39%	2009.8	4.17%
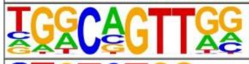	AMYB(HTH)/Testes-AMYB-ChIP-Seq(GSE44588)/Homer	1e-4	-9.920e+00	0.0007	92.0	19.78%	6359.0	13.18%
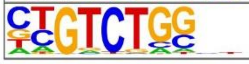	Smad2(MAD)/ES-SMAD2-ChIP-Seq(GSE29422)/Homer	1e-4	-9.853e+00	0.0007	110.0	23.66%	7968.3	16.52%

Motif enrichment in G12Vp53 ChIP peaks

Total Target Sequences = 2866, Total Background Sequences (genome) = 45361

Motif	Name	P-value	log P-pvalue	q-value (Benjamini)	# Target Sequences with Motif	% of Targets Sequences with Motif	# Background Sequences with Motif	% of Background Sequences with Motif
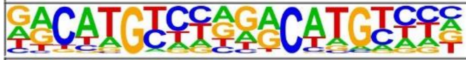	p53(p53)/Saos-p53-ChIP-Seq(GSE15780)/Homer	1e-2903	-6.685e+03	0.0000	2316.0	80.81%	440.0	0.97%
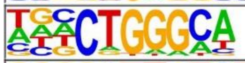	ZNF416(Zf)/HEK293-ZNF416.GFP-ChIP-Seq(GSE58341)/Homer	1e-186	-4.290e+02	0.0000	1285.0	44.84%	8973.8	19.81%
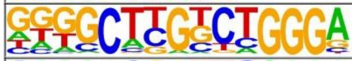	Zfp809(Zf)/ES-Zfp809-ChIP-Seq(GSE70799)/Homer	1e-97	-2.244e+02	0.0000	303.0	10.57%	993.5	2.19%
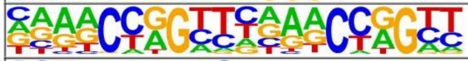	Tcfcp2l1(CP2)/mES-Tcfcp2l1-ChIP-Seq(GSE11431)/Homer	1e-85	-1.980e+02	0.0000	208.0	7.26%	498.3	1.10%
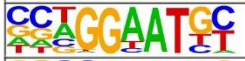	TEAD4(TEA)/Tropoblast-Tead4-ChIP-Seq(GSE37350)/Homer	1e-69	-1.601e+02	0.0000	564.0	19.68%	3892.9	8.59%
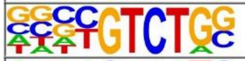	Smad4(MAD)/ESC-SMAD4-ChIP-Seq(GSE29422)/Homer	1e-62	-1.432e+02	0.0000	817.0	28.51%	7102.8	15.68%
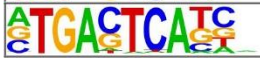	AP-1(bZIP)/ThioMac-PU.1-ChIP-Seq(GSE21512)/Homer	1e-47	-1.104e+02	0.0000	405.0	14.13%	2809.3	6.20%

Motif Enrichment in MCF10A-specific p53 ChIP peaks

Total Target Sequences = 565, Total Background Sequences (genome) = 46479

Motif	Name	P-value	log P-pvalue	q-value (Benjamini)	# Target Sequences with Motif	% of Targets Sequences with Motif	# Background Sequences with Motif	% of Background Sequences with Motif
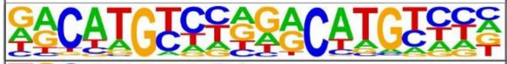	p53(p53)/Saos-p53-ChIP-Seq(GSE15780)/Homer	1e-871	-2.006e+03	0.0000	523.0	92.57%	523.2	1.11%
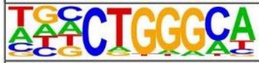	ZNF416(Zf)/HEK293-ZNF416.GFP-ChIP-Seq(GSE58341)/Homer	1e-34	-7.939e+01	0.0000	222.0	39.29%	8038.3	17.13%
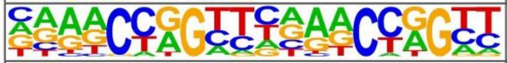	Tcfcp2l1(CP2)/mES-Tcfcp2l1-ChIP-Seq(GSE11431)/Homer	1e-27	-6.268e+01	0.0000	47.0	8.32%	446.9	0.95%
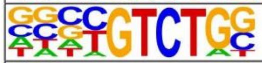	Smad4(MAD)/ESC-SMAD4-ChIP-Seq(GSE29422)/Homer	1e-16	-3.792e+01	0.0000	165.0	29.20%	7121.3	15.18%
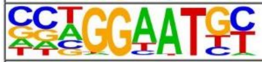	TEAD4(TEA)/Tropoblast-Tead4-ChIP-Seq(GSE37350)/Homer	1e-13	-3.223e+01	0.0000	109.0	19.29%	4115.8	8.77%
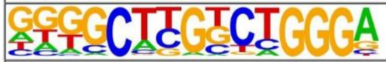	Zfp809(Zf)/ES-Zfp809-ChIP-Seq(GSE70799)/Homer	1e-11	-2.580e+01	0.0000	35.0	6.19%	700.6	1.49%
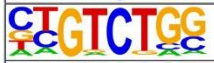	Smad2(MAD)/ES-SMAD2-ChIP-Seq(GSE29422)/Homer	1e-9	-2.267e+01	0.0000	142.0	25.13%	6954.1	14.82%
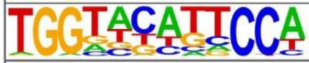	PRDM10(Zf)/HEK293-PRDM10.eGFP-ChIP-Seq(Encode)/Homer	1e-4	-1.108e+01	0.0005	64.0	11.33%	3046.5	6.49%
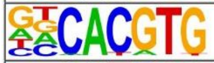	BMAL1(bHLH)/Liver-Bmal1-ChIP-Seq(GSE39860)/Homer	1e-4	-1.102e+01	0.0005	162.0	28.67%	9922.2	21.14%
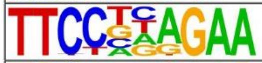	STAT6(Stat)/Macrophage-Stat6-ChIP-Seq(GSE38377)/Homer	1e-4	-1.008e+01	0.0010	50.0	8.85%	2264.5	4.83%
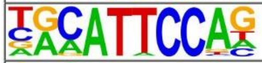	TEAD3(TEA)/HepG2-TEAD3-ChIP-Seq(Encode)/Homer	1e-4	-9.612e+00	0.0016	98.0	17.35%	5517.0	11.76%
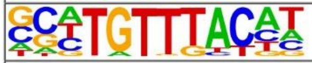	FOXK2(Forkhead)/U2OS-FOXK2-ChIP-Seq(E-MTAB-2204)/Homer	1e-3	-7.545e+00	0.0116	58.0	10.27%	3057.9	6.52%
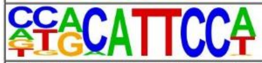	TEAD1(TEAD)/HepG2-TEAD1-ChIP-Seq(Encode)/Homer	1e-3	-7.028e+00	0.0185	78.0	13.81%	4511.4	9.61%
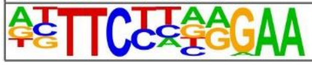	STAT6(Stat)/CD4-Stat6-ChIP-Seq(GSE22104)/Homer	1e-3	-6.911e+00	0.0197	45.0	7.96%	2275.0	4.85%

Supplementary Figure S7. Motif enrichment in cell type-specific p53 binding sites.

Enrichment was calculated in G12V-specific, the whole G12V, and MCF10A-specific p53 binding loci (ChIP peaks) against the genomic sequence background.

Supplementary Table S1. Primers used for measuring gene expression by real time PCR.

Gene	Primer 5' to 3'	
**SDC1**	Forward	TCCCCACACAGAGGATGGAG
	Reverse	CTCCCCCGAGGTTTCAAAGG
**NDRG**	Forward	GTACTTCGTGCAGGGCATGG
	Reverse	GCGGGTGCCATCCAGAGAA
**COL17A1**	Forward	CTCCCCATCCCCAAGAAAGGC
	Reverse	GACCACACATGGGAGGGAAGG
**TP63**	Forward	GTCATTTGATTCGAGTAGAGGGG
	Reverse	CTGGGGTGGCTCATAAGGT
**SERPINB5**	Forward	CGCAATGGATGCCCTGCAAC
	Reverse	AGGACATTGCCCAGTGGCTC
**EPS8L2**	Forward	CACACTGAGGTCTGCCCTTC
	Reverse	GTCCTTGGGGCTCATCTTGG
**EFNB1**	Forward	CCGCACACGCACCATGAAG
	Reverse	GCCTGTGTGGCCATCTTGAC
**TP53INP1**	Forward	GCCCAAGTAGTCCCAGAGTGG
	Reverse	CCACTGGGAAGGGCGAAAG
**FAM198B**	Forward	AGGATCCTGGGGCTCAACAG
	Reverse	GCGCATGGAAAGACACGCTG
**RGS12**	Forward	CCGGTCAACATCGACAGCC
	Reverse	TGGTACAGCGGGGACTTCAG

Supplementary Table S2. Inverse primers used for 4C library amplification.

Inverse PCR primers for 4C		
**Bait**	**Forward primer**	**Reverse primer**
FN1	CCTGTGGCAATTACTGAAGGTG	GCAAGATAAACCTGTAGCTAAAGC
CDH1	GCAGGGGCTAGAAACAAGC	ATTGTCACAACGATAAGGCC
FBN1	TGCCACCTTTCAGAGACCAT	GGCATCCTGTTAAGCAACAAAG
TP53	CAGCTGAGAGCAAACGCAAA	GGCTTTCCGTAATATCACACCC
MMP1	CCATAAACAAGTCATTCTCAAAGC	ACTAAAGCCAAGATTTCTGTTACTG
