# Supplementary material for: Genomic retargeting of p53 and CTCF is associated with transcriptional changes during oncogenic HRas-induced transformation
Source: Commun Biol. 2020 Nov 25;3:696. doi: 10.1038/s42003-020-01398-y (PMC7809021; doi:10.1038/s42003-020-01398-y)
Supplement: Supplementary file 3 — Description of Additional Supplementary Files [file 42003_2020_1398_MOESM3_ESM.pdf]

Description of Additional Supplementary Files

Supplementary Data 1: Differentially expressed genes. Differential gene expression between MCF10A and G12V cells based on two replicas of RNA-seq. First five columns indicate gene annotation- genomic location (chromosome, strand, transcription start and end site) and name. Next 4 columns indicate the CPM value in the two cell types in two replicas. The last three columns indicate log2 fold change, p Value and the direction of change: 1 and (-1) are up-regulated and down-regulated in G12V MCF10A cells respectively.

Supplementary Data 2: Functions of Diseases enriched in down-regulated genes associated with G12V-specific p53 binding sites (blue) or linked to up-regulated genes associated with G12V-specific p53 binding sites from IPA analysis. Functions with a log10 p >3 are shown. Functions of Biological Process enriched in G12V-specific p53 binding sites (blue) or linked to G12V-specific p53 binding sites from GREAT analysis.

Supplementary Data 3: Source Data file.
