# Supplementary material for: Genomic retargeting of p53 and CTCF is associated with transcriptional changes during oncogenic HRas-induced transformation
Source: Commun Biol. 2020 Nov 25;3:696. doi: 10.1038/s42003-020-01398-y (PMC7809021; doi:10.1038/s42003-020-01398-y)
Supplement: Supplementary file 7 — Reporting Summary [file 42003_2020_1398_MOESM7_ESM.pdf]

nature research

Corresponding author(s): Ofir Hakim

Last updated by author(s): 31.8.20

Reporting Summary

Nature Research wishes to improve the reproducibility of the work that we publish. This form provides structure for consistency and transparency in reporting. For further information on Nature Research policies, see Authors & Referees and the Editorial Policy Checklist .

Statistics

For all statistical analyses, confirm that the following items are present in the figure legend, table legend, main text, or Methods section.

n/a	Confirmed	
☐	☒	The exact sample size (*n*) for each experimental group/condition, given as a discrete number and unit of measurement
☐	☒	A statement on whether measurements were taken from distinct samples or whether the same sample was measured repeatedly
☐	☒	The statistical test(s) used AND whether they are one- or two-sided<br>*Only common tests should be described solely by name; describe more complex techniques in the Methods section.*
☐	☒	A description of all covariates tested
☐	☒	A description of any assumptions or corrections, such as tests of normality and adjustment for multiple comparisons
☐	☒	A full description of the statistical parameters including central tendency (e.g. means) or other basic estimates (e.g. regression coefficient) AND variation (e.g. standard deviation) or associated estimates of uncertainty (e.g. confidence intervals)
☐	☒	For null hypothesis testing, the test statistic (e.g. *F*, *t*, *r*) with confidence intervals, effect sizes, degrees of freedom and *P* value noted<br>*Give P values as exact values whenever suitable.*
☒	☐	For Bayesian analysis, information on the choice of priors and Markov chain Monte Carlo settings
☒	☐	For hierarchical and complex designs, identification of the appropriate level for tests and full reporting of outcomes
☒	☐	Estimates of effect sizes (e.g. Cohen's *d*, Pearson's *r*), indicating how they were calculated

Our web collection on statistics for biologists contains articles on many of the points above.

Software and code

Policy information about availability of computer code

Data collection	No software was used
Data analysis	ATAC seq analysis – Reads were mapped to the genome using bowtie v0.12.9. Regulatory regions were called using MACS v1.4.2 with the following parameters - --tsize=51 --nomodel --shiftsize=75 --llocal=25000 -p 1e-04.<br>RNA seq analysis – Reads were mapped to the genome using TopHat2 v2.1.1. Reads on genes were counted using HTSeq v0.6.0, htseq-count. For DE analysis edgeR v3.22.5 was used.<br>Ingenuity (IPA) was used for pathway and functional analysis.

For manuscripts utilizing custom algorithms or software that are central to the research but not yet described in published literature, software must be made available to editors/reviewers. We strongly encourage code deposition in a community repository (e.g. GitHub). See the Nature Research guidelines for submitting code & software for further information.

Data

Policy information about availability of data

All manuscripts must include a data availability statement. This statement should provide the following information, where applicable:

- Accession codes, unique identifiers, or web links for publicly available datasets
- A list of figures that have associated raw data
- A description of any restrictions on data availability

The sequencing data has been deposited to the GEO database (accession number GSE140254)

Field-specific reporting

Please select the one below that is the best fit for your research. If you are not sure, read the appropriate sections before making your selection.

☒ Life sciences ☐ Behavioural & social sciences ☐ Ecological, evolutionary & environmental sciences

For a reference copy of the document with all sections, see nature.com/documents/nr-reporting-summary-flat.pdf

Life sciences study design

All studies must disclose on these points even when the disclosure is negative.

Sample size	NA
Data exclusions	No data were excluded from the analysis.
Replication	molecular experiments and genomics were replicated as indicated in the manuscript (3 replicates for qPCR, 2 for RNA-seq, 2 for ATAC-seq, 2 for ChIP-seq). All the results were included.
Randomization	In this study MCF10A human mammary epithelial cell line was transformed by G12V H-Ras oncogene. The parental and transformed cell lies were used for all the experiments described in this study.
Blinding	Given that this study was focused on two samples- normal and transformed cells, blinding was not necessary

Reporting for specific materials, systems and methods

We require information from authors about some types of materials, experimental systems and methods used in many studies. Here, indicate whether each material, system or method listed is relevant to your study. If you are not sure if a list item applies to your research, read the appropriate section before selecting a response.

Materials & experimental systems

n/a	Involved in the study	
☐	☒	Antibodies
☐	☒	Eukaryotic cell lines
☒	☐	Palaeontology
☐	☒	Animals and other organisms
☒	☐	Human research participants
☒	☐	Clinical data

Methods

n/a	Involved in the study	
☐	☒	ChIP-seq
☒	☐	Flow cytometry
☒	☐	MRI-based neuroimaging

Antibodies

Antibodies used	Mouse anti-p53 monoclonal antibody for ChIP, western blot and immunofluorescence - Santa Cruz Biotechnology , catalog number SC-126, clone DO-1, Lot # I2817.<br>Rabbit anti -CTCF for ChIP and western blot - Millipore, catalog number 07-729.<br>Rabbit anti-GAPDH for western blot - Cell Signaling Technology, catalog number cst-2118
Validation	NA

Eukaryotic cell lines

Policy information about cell lines

Cell line source(s)	MCF10A
Authentication	Authentication by bioSYNTHESIS show that the cells used in this study a fully (100%) match MCF10A. No contamination by another cell line was detected.
Mycoplasma contamination	All the cells were tested for mycoplasma using "EZ-PCR PCR Mycoplasma Test Kit" , Biological Industries, Cat. No.: 20-700-20
Commonly misidentified lines (See ICLAC register)	No misidentified lines were used.

Animals and other organisms

Policy information about studies involving animals; ARRIVE guidelines recommended for reporting animal research

Laboratory animals	6 weeks old non-obese diabetic/severe combined immunodeficient (NOD/SCID) mice
Wild animals	The study did not involve wild animals.
Field-collected samples	The study did not involve samples collected from the field.
Ethics oversight	All studies with mice were approved by Institutional Animal Care and Use Committee at the Hebrew University of Jerusalem

Note that full information on the approval of the study protocol must also be provided in the manuscript.

ChIP-seq

Data deposition

☒ Confirm that both raw and final processed data have been deposited in a public database such as GEO.

☒ Confirm that you have deposited or provided access to graph files (e.g. BED files) for the called peaks.

Data access links
May remain private before publication.

To review GEO accession GSE140254:
https://eur02.safelinks.protection.outlook.com/?url=https%3A%2F%2Fwww.ncbi.nlm.nih.gov%2Fgeo%2Fquery%2Facc.cgi%3Facc%3DGSE140254&data=02%7C01%7Cavital.sarusi%40biu.ac.il%7C567b68b1e3fe462343c208d76859e8c2%7C61234e145b874b67ac198feaa8ba8f12%7C0%7C0%7C637092607703499784&sdata=JKNS7zdU50GVn5WutcRAQXOAG4I2z5Srs4YAtKKHtEk%3D&reserved=0
Token qlgjaskwrjsrnap

Files in database submission

GSM4158364 MCF10A_ATAC_WT_1
GSM4158365 MCF10A_ATAC_WT_2
GSM4158366 MCF10A_ATAC_HRAS_1
GSM4158367 MCF10A_ATAC_HRAS_2
GSM4158368 MCF10A_WT_CTCF_1
GSM4158369 MCF10A_WT_input_CTCF_1
GSM4158370 MCF10A_WT_CTCF_2
GSM4158371 MCF10A_WT_input_CTCF_2
GSM4158372 MCF10A_HRAS_CTCF_1
GSM4158373 MCF10A_HRAS_input_CTCF_1
GSM4158374 MCF10A_HRAS_CTCF_2
GSM4158375 MCF10A_HRAS_input_CTCF_2
GSM4158376 MCF10A_WT_p53_1
GSM4158377 MCF10A_WT_input_p53_1
GSM4158378 MCF10A_WT_p53_2
GSM4158379 MCF10A_WT_input_p53_2
GSM4158380 MCF10A_HRAS_p53_1
GSM4158381 MCF10A_HRAS_input_p53_1
GSM4158382 MCF10A_HRAS_p53_2
GSM4158383 MCF10A_HRAS_input_p53_2
GSM4158384 MCF10A_Nut_RNA_WT_1 [WT_1_NUT]
GSM4158385 MCF10A_Nut_RNA_WT_2 [WT_2_NUT]
GSM4158386 MCF10A_Nut_RNA_WT_3 [WT_3_NUT]
GSM4158387 MCF10A_RNA_HRAS_1 [RAS_rep1]
GSM4158388 MCF10A_RNA_HRAS_2 [RAS_rep2]
GSM4158389 MCF10A_RNA_WT_1 [cont_rep1]
GSM4158390 MCF10A_RNA_WT_2 [cont_rep2]
GSM4158391 MCF10A_woNut_RNA_WT_1 [WT_1]
GSM4158392 MCF10A_woNut_RNA_WT_2 [WT_2]
GSM4158393 MCF10A_woNut_RNA_WT_3 [WT_3]

Genome browser session
(e.g. UCSC)

http://genome.ucsc.edu/s/Avital/MCF10A_ChIP_paper

Methodology

Replicates

for ChIP-seq Replicates – Two biological replicas per cell type. Replicas correlation was calculated using sampling 10M reads per replica, calling peaks and calculating the correlation between the merged peaks. Correlation was between 0.79-0.9.

Sequencing depth

Sequencing depth – All reads were single-end.
Sample Reads Aligned % Read length
MCF10A_WT_input_1 37,246,695 28,880,320 77.54% 80
MCF10A_WT_p53_1 28,492,938 22,149,896 77.74% 80
MCF10A_HRAS_input_1 13,896,241 11,144,535 80.20% 80
MCF10A_HRAS_p53_1 15,038,167 9,404,378 62.54% 80
MCF10A_WT_input_2 26,999,562 21,877,934 81.03% 80
MCF10A_WT_p53_2 26,772,803 21,016,344 78.50% 80
MCF10A_HRAS_input_2 30,826,880 24,773,772 80.36% 80
MCF10A_HRAS_p53_2 42,925,206 34,445,596 80.25% 80
MCF10A_WT_input_1 16,494,267 12,455,699 75.52% 60
MCF10A_WT_CTCF_1 24,463,929 19,082,671 78.00% 60
MCF10A_HRAS_input_1 18,376,774 13,992,063 76.14% 60
MCF10A_HRAS_CTCF_1 17,756,339 13,887,983 78.21% 60
MCF10A_WT_input_2 14,761,841 11,234,365 76.10% 60
MCF10A_WT_CTCF_2 15,113,327 11,801,018 78.08% 60
MCF10A_HRAS_input_2 14,554,389 11,062,624 76.01% 60
MCF10A_HRAS_CTCF_2 14,864,912 11,731,096 78.92% 60

Antibodies

Mouse anti-p53 monoclonal antibody for ChIP, western blot and immunofluorescence - Santa Cruz Biotechnology , catalog number SC-126, clone DO-1, Lot # l2817.
Rabbit anti -CTCF for ChIP and western blot - Millipore, catalog number 07-729.
Rabbit anti-GAPDH for western blot - Cell Signaling Technology, catalog number cst-2118

Peak calling parameters

Mapping – Bowtie was used with the following command for all samples -bowtie -m 1 -q –S, reads were aligned to the hg19 genome.
Peak calling – MACS2 was used with the following command for all samples - macs2 callpeak -t IP_SAMPLE -c INPUT_SAMPLE -g hs

Data quality

MACS2 default parameter is to call peaks with minimum FDR of 5%. Number of peaks is describe below. To assess the quality of the peaks, the number of reads covered within peaks was calculated for each sample. In addition, the overlap between the peaks and the motif of the factor, since we used known factors with known motifs, was also calculated.
Sample Peaks
MCF10A_WT_p53_1 1,718
MCF10A_HRAS_p53_1 477
MCF10A_WT_p53_2 885
MCF10A_HRAS_p53_2 2,619
MCF10A_WT_CTCF_1 11,346
MCF10A_HRAS_CTCF_1 12,004
MCF10A_WT_CTCF_2 20,136
MCF10A_HRAS_CTCF_2 27,678

Software

Bowtie was used to align the reads to the human genome. MACS2 was used for peak calling. For overlap between peaks, motifs, genes and so on bedtools IntersectBed was used. Homer was used to find motifs. R software was used for plotting. UCSC genome browser was used for visualization.
